# Supplementary figures and images for: NCAPG Promotes Pulmonary Artery Smooth Muscle Cell Proliferation as a Promising Therapeutic Target of Idiopathic Pulmonary Hypertension: Bioinformatics Analysis and Experiment Verification
Source: Int J Mol Sci. 2022 Oct 4;23(19):11762. doi: 10.3390/ijms231911762 (PMC9570379; doi:10.3390/ijms231911762)

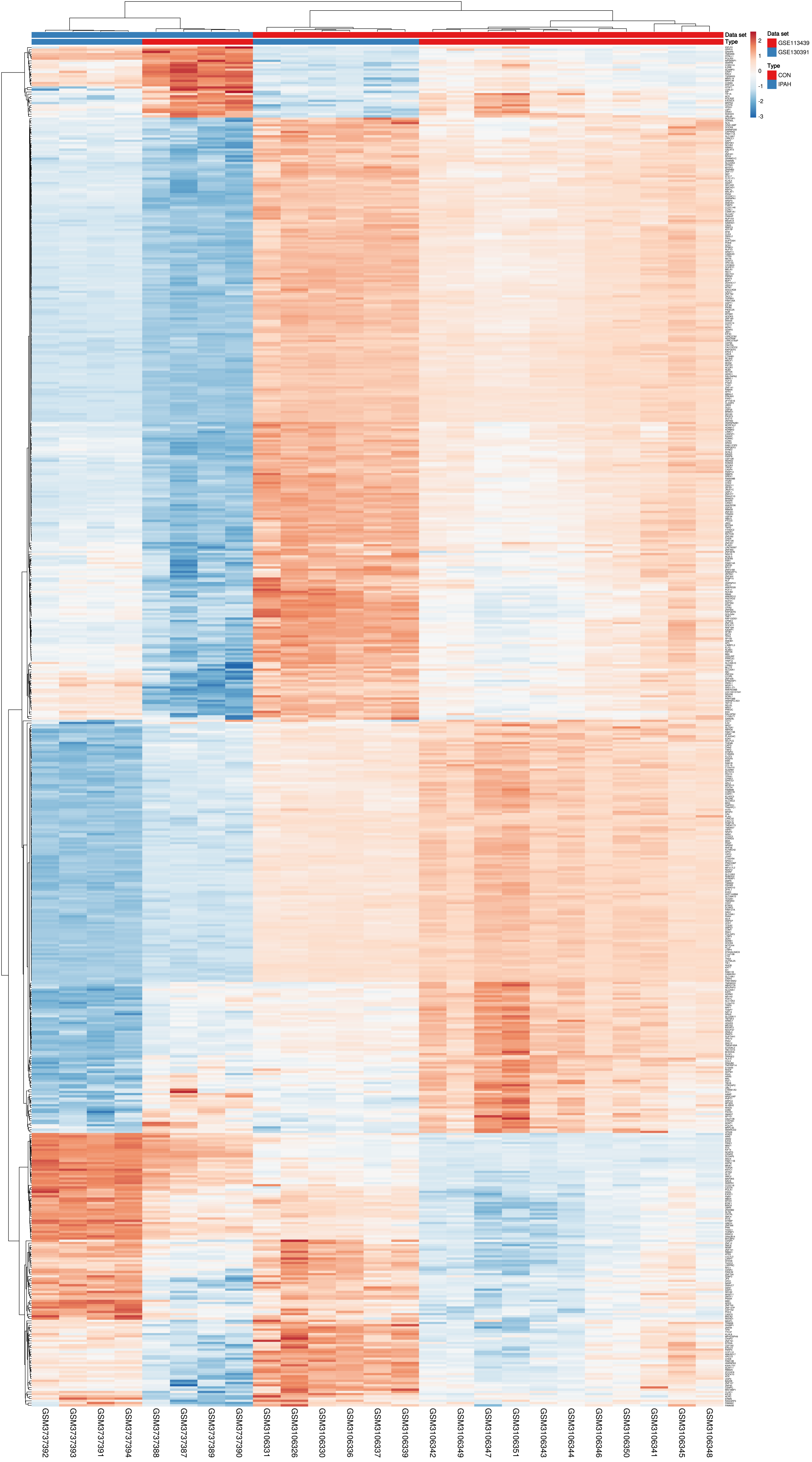

Supplement: Supplementary file 1 [file ijms-23-11762-s001.zip › Figure S1.pdf]

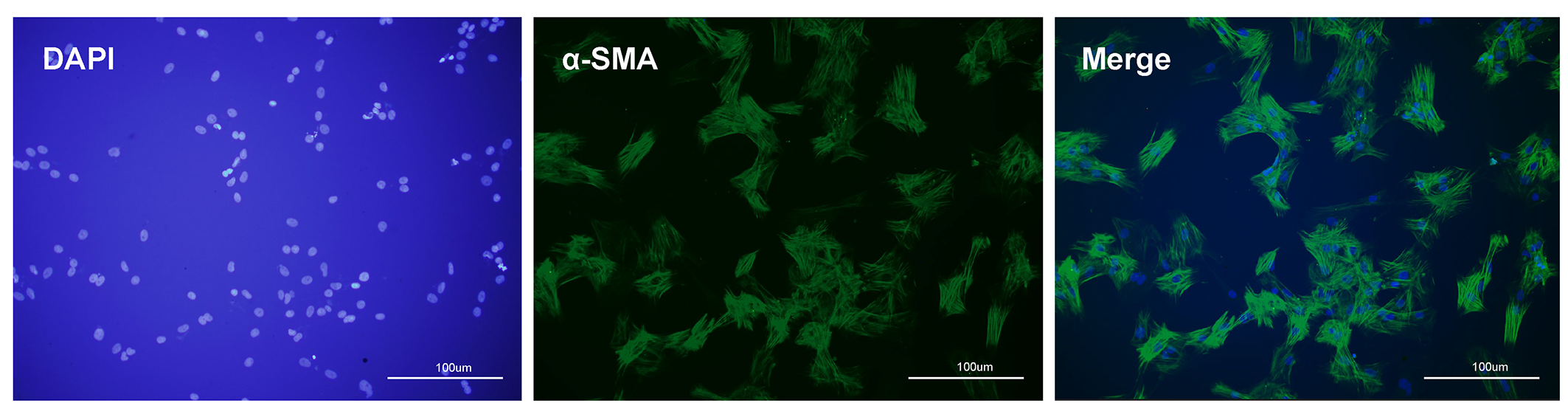

Supplement: Supplementary file 1 [file ijms-23-11762-s001.zip › Figure S2.tif]
